# Reach and engagement on the history of nursing on social media in light of Pierre Lévy

**DOI:** 10.1590/0034-7167-2024-0228

**Published:** 2025-01-13

**Authors:** Natália Maria Freitas e Silva Maia, Agostinho Antônio Cruz Araújo, Ana Maria Ribeiro dos Santos, Rafaela Siqueira Costa Schreck, Maria Angélica de Almeida Peres, Fernanda Batista Oliveira Santos

**Affiliations:** I Universidade Federal do Piauí. Teresina, Piauí, Brazil; II Universidade de São Paulo. Ribeirão Preto, São Paulo, Brazil; III Universidade Federal de Minas Gerais. Belo Horizonte, Minas Gerais, Brazil; IV Universidade Federal do Rio de Janeiro. Rio de Janeiro, Rio de Janeiro, Brazil

**Keywords:** History of Nursing, Internet, Media, Museums, Social Network, Historia de la Enfermería, Internet, Medios de Comunicación, Museos, Redes Sociales

## Abstract

**Objective:**

To analyze the reach and engagement on the history of nursing on social media of the Memory Center of the School of Nursing, Federal University of Minas Gerais (CEMENF/UFMG), in light of Pierre Lévy.

**Methods:**

Documentary study carried out on CEMENF’s Instagram and on the YouTube of the School of Nursing of UFMG, from September to December 2021. The findings were analyzed according to Pierre Lévy’s concepts.

**Results:**

Publications on social media, in the years 2020 and 2021, resulted in an average of 6.79 feed posts and 1.42 Reels on Instagram, with an average of 25.54 comments on feed posts and 512.17 views on Reels. As for YouTube, an average of 1.58 posts was observed, with 2164.08 views and 101.58 likes.

**Conclusions:**

Cyberspace can be a powerful instrument for bringing university museums closer to society and for disseminating extension and research projects.

## RESUMEN

**Objetivo:**

Analizar el alcance y el compromiso con la historia de la enfermería en las redes sociales del Centro de Memoria de la Escuela de Enfermería de la Universidad Federal de Minas Gerais (CEMENF/UFMG) a la luz de Pierre Lévy.

**Métodos:**

Se trata de un estudio documental del CEMENF realizado por Instagram y YouTube de la Escuela de Enfermería de la UFMG, entre septiembre y diciembre de 2021. Las deducciones se analizaron según los conceptos de Pierre Lévy.

**Resultados:**

Las publicaciones en redes sociales en 2020 y 2021 dieron lugar a una media de 6,79 feed posts y 1,42 Reels en Instagram, con una media de 25,54 comentarios en los feed posts y 512,17 visualizaciones en los Reels. Con relación a YouTube, hubo una media de 1,58 publicaciones con 2164,08 visualizaciones y 101,58 me gusta.

**Conclusiones:**

El ciberespacio puede ser una poderosa herramienta para acercar los museos universitarios a la sociedad y difundir proyectos de extensión e investigación.

## INTRODUCTION

The appropriation of technologies and cyberspace by society has become an increasingly growing necessity in today’s world and permeates all segments, namely: political, economic, social or cultural. Cyberspace is understood as an open space for communication, which arises from the global interconnection of computers^(1)^. The absence of physical limits, created by cyberspace, provides different forms of human experiences and social interaction, which also enables the construction and acquisition of knowledge. In this way, it can permeate the teaching-learning process, as evidenced by a critical review of the literature^(2)^. From this perspective, digital technologies are essential for the history of nursing in the training process of students^(3,4)^. Above all, because it is through this history that scientific support and professional practice are obtained, as well as the construction of their identity^(5)^.

In the historical context of nursing, memory centers gain prominence. These are spaces present in universities, schools, companies and public bodies that have the function of guarding, incorporating, preserving, researching and communicating institutional history, combining knowledge and characteristics specific to museums, archives and libraries. In a dialogic way with the public, they exhibit the material and immaterial testimonies of people and their environments for the purposes of study, education and leisure, in a given historical period^(6)^.

From the academic and professional perspective of Brazilian nursing, the first initiatives to create these places of memory date back to the 1970s and 1980s. For example, institutions became aware of the production, recovery and preservation of historical sources and nursing memories, which motivated the creation of groups and lines of research in postgraduate programs, holding events and publishing in scientific journals^(7)^.

The Federal University of Minas Gerais (UFMG) has a Network of Museums and Spaces for Science and Culture^(8)^, in which the Memory Center of the School of Nursing (CEMENF) has participated since its creation in 2006. In January 2019, CEMENF/UFMG began work to review its social media, with planning and investment in museum communication, through the diversification of teaching, research and extension activities to reach and engage the university public, as well as new audiences, and to expand access to the university by society in general.

The use of social media and museum communication strategies requires an understanding of the concepts of reach and engagement, which are part of the structure of these media. Reach is defined as the visibility capacity that content obtains for a target audience, that is, the number of accesses, views and followers of a given profile or virtual account^(9)^. Engagement, on the other hand, is understood from the public’s interactions with the content and occurs through likes, comments and shares^(10)^.

It is worth noting that the resources available on social networks direct the process of interaction and content generation, bringing users closer to pages of their social interest, to obtain information and interact with what is published^(10)^. Given this premise, the use of social media can contribute to the acquisition of historical knowledge. Furthermore, the reach and engagement of CEMENF/UFMG on social media can signal how much it has been fulfilling its role of valuing, rescuing and popularizing historical sources and nursing memory.

## OBJECTIVE

To analyze reach and engagement on the history of nursing on social media at the Memory Center of the School of Nursing, Federal University of Minas Gerais (CEMENF/UFMG), in light of Pierre Lévy.

## METHODS

### Ethical aspects

As this is secondary data, the research does not require approval from the Research Ethics Committee.

### Design and setting

This is a documentary research carried out at the Memory Center of the School of Nursing, Federal University of Minas Gerais (CEMENF/UFMG). CEMENF is a museum space for documentation and research on the memory and history of nursing and health in Minas Gerais, Brazil, which seeks dialogue between the university and society through the articulation of teaching, research and extension activities^(11)^. The space works to preserve and encourage the study of the School’s collection, which is organized, catalogued and open to the public^(12)^.

### Study Protocol

The documents that supported this study were produced by the team of the extension project “Scientific Dissemination and Museological Communication: Virtual Platform for Visits and Digital Productions”. The objective of this project was to virtualize content produced by the museum team and make it accessible to the public in cyberspace. Data collection took place on the CEMENF/UFMG Instagram (@ufmgcemenf) and on the YouTube of the UFMG School of Nursing (@ufmgenfermagem), from September to December 2021, the month in which the project was completed.

The data were collected using a semi-structured form, in order to include the total number of views of the content published on Instagram and YouTube, in the years 2020 and 2021, corresponding to the period in which remote activities were at their peak due to the COVID-19 pandemic. In these years, there was greater use of social media due to the determination of the Ministry of Health for the population to maintain social distancing. Furthermore, nursing history themes were disseminated worldwide in 2020, the year of the bicentennial celebration of Florence Nightingale, which justifies the time frame of this study.

### Analysis of results and statistics

The information was tabulated and organized in Microsoft Excel 2016®, and statistically analyzed in the Statistical Package for the Social Sciences (SPSS) - Version 26. The findings were presented in graphs and tables, which were analyzed according to the concepts of cyberspace, interconnection, virtual communities and social intelligence, all by Pierre Lévy. It is worth noting that interconnection is understood from a borderless continuum, enabling the diversity of interactive communication. Virtual communities, in turn, “are built on affinities of interests, knowledge, mutual projects, in a process of cooperation or exchange, all of this regardless of geographic proximity and institutional affiliations”. Finally, collective intelligence improves knowledge and accelerates the learning of its members, enhancing the development of collective intelligence, which “would be its spiritual perspective, its ultimate purpose”^(13)^.

## RESULTS

The publications on the social networks of the Memory Center of the UFMG School of Nursing resulted in an average of 6.79 feed posts and 1.42 Reels on Instagram, data referring to the years 2020 and 2021. For this same two-year period, an average of 25.54 comments on the feed posts and 512.17 views on the Reels were obtained. As for YouTube, an average of 1.58 posts was observed, with 2164.08 views and 101.58 likes (Table 1).

**Table 1:** Characterization of Publications Made by the Memory Center of the School of Nursing of Federal University of Minas Gerais, Belo Horizonte, Minas Gerais, Brazil, 2021

	Mean (CI-95%)	SD	*p* value^*^
Instagram - Feed			
Number of posts	6.79(4.55-9.04)	5.32	0.398
Number of likes	268.50(177.52-359.48)	215.45	0.268
Number of comments	25.54(16.57-34.52)	21.25	0.209
Instagram - Reels			
Number of posts	1.42(0.51-2.32)	2.15	0.001
Number of views	512.75(93.95-931.55)	991.80	0.001
Number of comments	11.04(1.71-20.37)	22.10	<0.001
Youtube			
Number of Posts	1.58(0.52-2.65)	1.68	0.062
Number of Views	2164.08(-592.88-4921.05)	4339.15	<0.001
Number of Comments	3.33(-1.00-7.66)	6.81	<0.001
Likes	101.58(-12.71-215.87)	179.88	<0.001

* *Shapiro-Wilk test, at the 5% level.*

**Figure 1:**
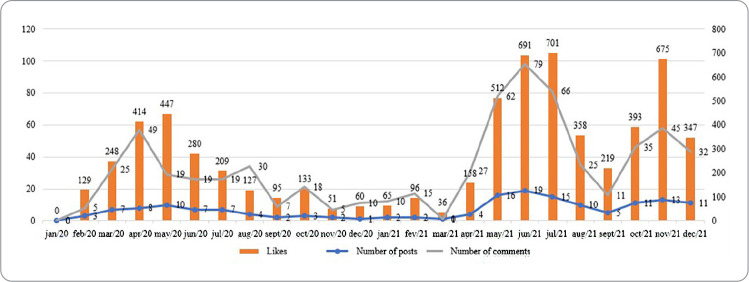
Trajectory between the counts of Instagram feed posts that were carried out by the Memory Center of the Federal University of Minas Gerais School of Nursing, Belo Horizonte, Minas Gerais, Brazil, 2021

It can be seen that the number of feed posts produced and published on Instagram throughout 2020 and 2021 was not uniform and varied throughout the months. The highest count was seen between May and July 2021. During this same period, there was an increase in the number of likes and comments (Figure 1).

In the bivariate analysis, Pearson’s correlation coefficient indicated that likes and comments were positively correlated with feed posts, r = 0.958 and r = 0.905, respectively.

**Figure 2:**
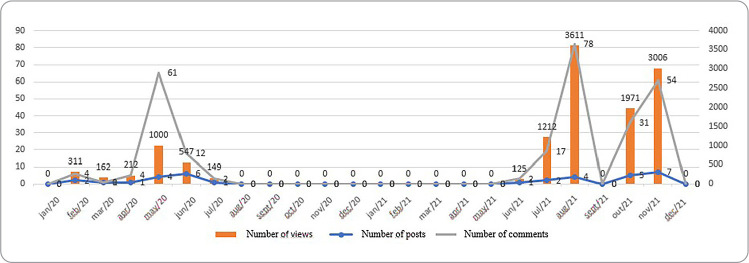
Analysis of the trajectory between the counts of Instagram-Reels posts carried out by the Memory Center of the School of Nursing of Federal University of Minas Gerais, Belo Horizonte, Minas Gerais, Brazil, 2021

As for Instagram Reels posts, it was observed that there was no production between August 2020 and May 2021. November 2021 was the month with the highest number of posts (7), however August 2021 was the month with the highest number of views (3611) and comments (78) (Figure 2). Pearson’s correlation coefficient was positive between Reels posts and views (r = 0.979), as well as comments (r=0.940).

**Figure 3:**
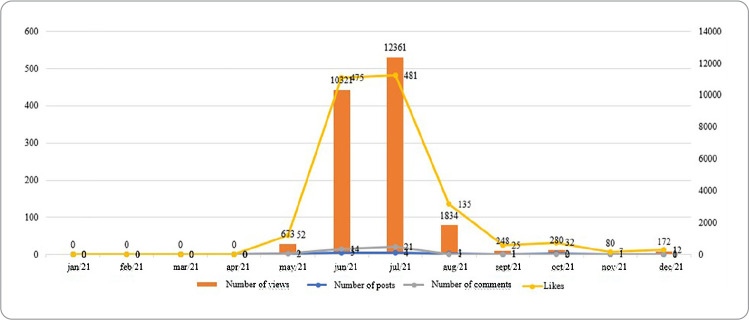
Analysis of the trajectory between the counts of Youtube posts carried out by the Memory Center of the School of Nursing of Federal University of Minas Gerais, Belo Horizonte, Minas Gerais, Brazil, 2021

In the meantime, the videos produced by CEMENF for YouTube were produced in greater numbers in June 2021 (5), however, in July 2021, they had a higher number of views (12,361), comments (21) and likes (481) (Figure 3). YouTube videos had a positive correlation with views (r=0.984), comments (r=0.802) and likes (r=0.984).

**Figure 4:**
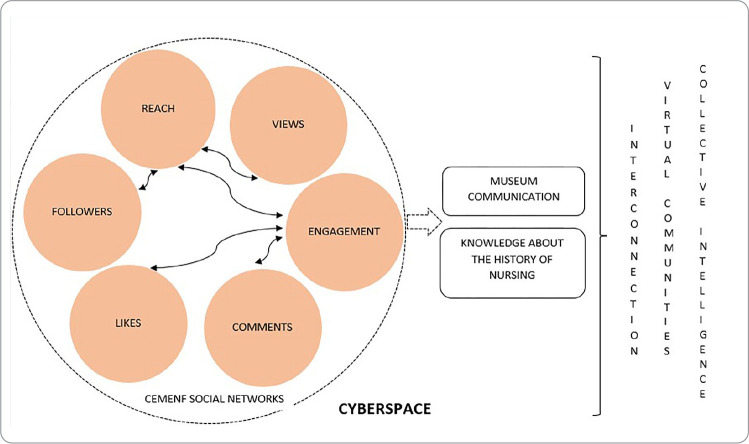
Representation of the conformation of Memory Center of the School of Nursing of Federal University of Minas Gerais social networks in cyberspace, Belo Horizonte, Minas Gerais, Brazil, 2021

Therefore, the evidence made it possible to build an image that represents the configuration of CEMENF’s social networks in cyberspace, in light of Pierre Levy’s concepts (Figure 4).

## DISCUSSION

The creation and dissemination of historical nursing content on social media contributes to the establishment of CEMENF in cyberspace, understood from the perspective of interactive, reciprocal, community and intercommunity communication practices^(13)^. This establishment was achieved through the diversity of publications produced by CEMENF on its different social networks, through feed and Reels posts on Instagram and videos on YouTube.

It is clear that the reach and engagement obtained with the publications, through views, likes and comments, constitute cyberspace as understood by Pierre Levy^(13)^, who describes it as a living, heterogeneous virtual world in which each person can participate and contribute. Being part of this cyberspace reaffirms CEMENF’s commitment and challenge not only with the field of museological communication, but also with sharing the history of nursing, whether local, national or international, and beyond institutional walls. It is worth emphasizing that the use of cyberspace in the educational field can occur through the use of digital educational technologies. These technologies, when used in teaching nursing history, encourage learning and reconfigure this teaching^(14)^. It is worth noting that CEMENF’s social networks become an option for an interactive field of historical learning and asynchronous communication. Furthermore, they break with the more traditional ways of presenting and socializing historical content, which may be invisible to the current generation of students^(14,15)^. In the context of breaking with traditional methods, museums and memory centers present themselves as spaces for an active teaching-learning process through the articulation of historical and current facts, enabling critical reflection, as well as the strengthening and development of professional identity^(16)^. Thus, CEMENF’s social networks are means of giving visibility to this space, formed by a historical building, a collection of clothing, documentary and iconographic collections, among others. Furthermore, it is believed that it contributes to the awakening of the desire to know it, whether through a physical or virtual visit, called a guided tour.

In fact, it is observed that the publications on Instagram, in feeds and Reels, and on YouTube, although they present variations throughout the months in the period studied, reveal a positive correlation with likes, comments and views. This variation is due to the fact that all the content production is done by undergraduate and graduate nursing students, who combine the construction of this content on museological communication and the history of nursing with academic curricular activities.

Despite this, the data indicate that CEMENF’s social networks maintain interconnection, establishing contact and interactive communication with the users of these networks and, thus, building themselves as an engaging space for interaction on the history of nursing in Minas Gerais, Brazil and internationally. Therefore, interconnection is one of the principles that guided the growth of cyberspace and it is this that conceives a global contact^(13)^.

Furthermore, as the data presented indicate the reach and engagement of publications on CEMENF’s social networks, the creation of virtual communities is outlined, which share knowledge and impressions about common interests, in this case, historical knowledge of nursing. This possibility of aggregating and favoring interactions arising from publications brings together people who, according to Levy, were previously isolated or without regular contact and who now enjoy a continuous space for meeting and exchanges^(13)^. In addition, they encourage the improvement of CEMENF’s museological communication with the public. The philosopher Pierre Lévy conceptualizes collective intelligence as “[...] an intelligence distributed everywhere, incessantly valued, coordinated in real time, which results in an effective mobilization of skills”^(1)^. The development of such skills and abilities in individuals through the use of information and communication technologies, and when used for the benefit of the community, reflects their essence and relevance^(1)^. In this sense, the use of cyberspace by CEMENF can be considered an innovative strategy for the construction and production of knowledge specific to nursing (and health), since it represents a restructuring of teaching and the way of learning for the nursing class.

Consequently, CEMENF, by exploring cyberspace as a tool for reaching the public, socializes the history of nursing, in addition to demonstrating the relevance and scientific nature of nursing. Thus, it aims to contribute to the formation of critical subjects, aware of the history of their profession, endowed with a solid identity and capable of acting in favor of the social valorization of nursing.

To this end, CEMENF presents as a challenge the constancy in the production and publication of content for its social networks, in order to establish itself as a continuous and permanent space for communication and knowledge about the history of nursing, favoring interconnection, its consolidation as a virtual community and the construction of collective intelligence in this broad space, cyberspace. In this way, the essential interconnections that underpin cyberspace are reiterated in order to make museum communication viable in the current era of media communication.

### Study limitations

CEMENF’s Instagram account is a business account and its YouTube videos are hosted on the UFMG School of Nursing channel. Despite this, the study was limited by the fact that the metrics were not monitored more accurately by the Professional Panel, formerly Instagram Insights and YouTube Analytics. This monitoring would allow us to verify the audience profile and the main insights about the accounts reached.

### Contributions to the Area

Cyberspace, when used critically and reflectively, can be a powerful instrument for bringing university museums closer to society and for disseminating extension and research projects. In this way, this study contributes as an incentive for other Memory Centers to objectively outline alternatives and paths in favor of increasing the possibilities of access to culture and information, valuing nursing as a science and profession.

## CONCLUSIONS

In summary, the study shows how cyberspace can be configured as an important tool to enhance the reach and engagement of diverse audiences in culture and health knowledge, in addition to opening opportunities for fruitful dialogue between different types of knowledge, considering them and adding them together for a dialogical construction of collective intelligence, as proposed by Pierre Lévy.
