# Supplementary material for: Reach and engagement on the history of nursing on social media in light of Pierre Lévy
Source: Rev Bras Enferm. 2025 Jan 13;78(1):e20240228. doi: 10.1590/0034-7167-2024-0228 (PMC11731849; doi:10.1590/0034-7167-2024-0228)
Supplement: Translation

# Translation: Alcance e engajamento sobre a história da enfermagem nas mídias sociais à luz de Pierre Lévy

**DOI:** 10.1590/0034-7167-2024-0228pt

**Authors:** Natália Maria Freitas e Silva Maia, Agostinho Antônio Cruz Araújo, Ana Maria Ribeiro dos Santos, Rafaela Siqueira Costa Schreck, Maria Angélica de Almeida Peres, Fernanda Batista Oliveira Santos

**Affiliations:** I Universidade Federal do Piauí. Teresina, Piauí, Brasil; II Universidade de São Paulo. Ribeirão Preto, São Paulo, Brasil; III Universidade Federal de Minas Gerais. Belo Horizonte, Minas Gerais, Brasil; IV Universidade Federal do Rio de Janeiro. Rio de Janeiro, Rio de Janeiro, Brasil

**Keywords:** História da Enfermagem, Internet, Meios de Comunicação, Museus, Rede Social

## Abstract

**Objetivo:**

Analisar o alcance e o engajamento sobre a história da enfermagem nas mídias sociais do Centro de Memória da Escola de Enfermagem, da Universidade Federal de Minas Gerais (CEMENF/UFMG), à luz de Pierre Lévy.

**Métodos:**

Estudo documental realizado no Instagram do CEMENF e no YouTube da Escola de Enfermagem da UFMG, de setembro a dezembro de 2021. Os achados foram analisados segundo os conceitos de Pierre Lévy.

**Resultados:**

As publicações nas redes sociais, nos anos de 2020 e 2021, resultaram em uma média de 6,79 postagens de feed e 1,42 Reels no Instagram, com média de 25,54 comentários nas postagens de feed e 512,17 visualizações nos Reels. Quanto ao Youtube, observa-se uma média de 1,58 postagens, com 2164,08 visualizações e 101,58 curtidas.

**Conclusões:**

O Ciberespaço pode ser um potente instrumento de aproximação de museus universitários com a sociedade e de difusão de projetos de extensão e de pesquisa.

## INTRODUÇÃO

A apropriação das tecnologias e do ciberespaço pela sociedade têm se tornado uma necessidade cada vez mais crescente no mundo atual e perpassa todos os segmentos, sejam eles: políticos, econômicos, sociais e culturais. O ciberespaço é compreendido como um espaço aberto de comunicação, que surge da interconexão mundial dos computadores^(1)^. A ausência de limites físicos, criada pelo ciberespaço, favorece formas diferentes de vivências humanas e de interação social, o que também possibilita a construção e a apreensão do conhecimento. Desse modo, pode permear o processo de ensino-aprendizagem, assim como evidenciado por revisão crítica da literatura^(2)^. Nessa perspectiva, é fundamental ressaltar as tecnologias digitais para a história da enfermagem no processo formativo de estudantes^(3,4)^. Sobretudo porque é por meio dessa história que se tem a sustentação científica e da prática profissional, bem como a construção de sua identidade^(5)^.

No contexto histórico da enfermagem, os centros de memórias ganham destaque. Constituem-se de espaços presentes em universidades, escolas, empresas e órgãos públicos que têm como função a guarda, a incorporação, a preservação, a pesquisa e a comunicação da história institucional, mesclando conhecimentos e características próprias de museus, arquivos e bibliotecas. De forma dialógica com o público, expõem os testemunhos materiais e imateriais dos povos e seus ambientes para fins de estudo, educação e lazer, em determinado tempo histórico^(6)^.

Na perspectiva acadêmica e profissional da enfermagem brasileira, as primeiras iniciativas para a criação desses lugares de memória datam das décadas de 1970 e 1980. A exemplo disso, as instituições despertaram para a produção, a recuperação e a preservação de fontes históricas e de memória da enfermagem, o que motivou a criação de grupos e linhas de pesquisas nos programas de pós-graduação, realização de eventos e publicações em periódicos científicos^(7)^.

A Universidade Federal de Minas Gerais (UFMG) possui uma Rede de Museus e Espaços de Ciências e Cultura^(8)^, na qual o Centro de Memória da Escola de Enfermagem (CEMENF) participa desde a sua criação, em 2006. Em janeiro de 2019, o CEMENF/UFMG iniciou um trabalho de revisão de suas mídias sociais, com planejamento e investimento na comunicação museológica, mediante a diversificação de atividades de ensino, pesquisa e extensão para o alcance e o engajamento do público universitário, além de novos públicos, e a ampliação do acesso à universidade pela sociedade em geral.

O uso das mídias sociais e das estratégias de comunicação museológica requer a compreensão do conceito de alcance e engajamento, que são estruturantes nesses meios. O alcance é definido como a capacidade de visibilidade que um conteúdo obtém por um público-alvo, ou seja, quantidade de acessos, número de visualizações e seguidores de determinado perfil ou conta virtual^(9)^. Já o engajamento é compreendido a partir das interações do público com os conteúdos e ocorre pelas curtidas, comentários e compartilhamentos^(10)^.

Vale ressaltar que os recursos disponíveis nas redes sociais direcionam o processo de interação e geração de conteúdos, aproximando os usuários às páginas de seu interesse social, para obtenção de informações e interação com o que é publicado^(10)^. Diante dessa premissa, o uso das mídias sociais pode contribuir para a aquisição de conhecimentos históricos. Ademais, o alcance e o engajamento do CEMENF/UFMG nas mídias sociais podem sinalizar o quanto este vem cumprindo com o seu papel de valorização, resgate e popularização das fontes históricas e memória da enfermagem.

## OBJETIVO

Analisar o alcance e o engajamento sobre a história da enfermagem nas mídias sociais do Centro de Memória da Escola de Enfermagem, da Universidade Federal de Minas Gerais (CEMENF/UFMG), à luz de Pierre Lévy.

## MÉTODOS

### Aspectos éticos

Por se tratar de dados secundários, a pesquisa dispensa aprovação no Comitê de Ética em Pesquisa.

### Desenho e local

Trata-se de uma pesquisa documental, realizada no Centro de Memória da Escola de Enfermagem, da Universidade Federal de Minas Gerais (CEMENF/UFMG). O CEMENF é um espaço museográfico de documentação e pesquisa sobre a memória e a história da enfermagem e saúde em Minas Gerais, Brasil, que busca a interlocução entre a universidade e a sociedade por meio da articulação entre atividades de ensino, pesquisa e extensão^(11)^. O espaço atua na preservação e no estímulo ao estudo do acervo da Escola, que se encontra organizado, catalogado e aberto ao público^(12)^.

### Protocolo do estudo

Os documentos que subsidiaram este estudo foram produzidos pela equipe do projeto de extensão “Divulgação Científica e Comunicação Museológica: Plataforma Virtual para Visita e Produções Digitais”. Este teve como objetivo virtualizar conteúdos produzidos pela equipe do espaço museal e socializá-los de forma acessível ao público no ciberespaço. A coleta de dados ocorreu no Instagram do CEMENF/UFMG (@ufmgcemenf) e no YouTube da Escola de Enfermagem da UFMG (@ufmgenfermagem), no período de setembro a dezembro de 2021, mês de conclusão do projeto.

Os dados foram coletados por formulário semiestruturado, de modo a contemplar o total de visualizações dos conteúdos publicados no Instagram e YouTube, nos anos de 2020 e 2021, correspondentes ao período em que as atividades remotas estavam no auge em virtude da pandemia COVID-19. Nesses anos houveram maior utilização das mídias sociais devido à determinação do Ministério da Saúde para que a população mantivesse o afastamento social. Além disso, os temas de história da enfermagem foram mundialmente difundidos em 2020, ano de comemoração do bicentenário de Florence Nightingale, o que justifica o recorte temporal deste estudo.

### Análise dos resultados e estatística

As informações foram tabuladas e organizadas em Microsoft Excel 2016®, e analisadas estatisticamente no *Statistical Package for the Social Sciences* (SPSS) - Versão 26. Os achados foram apresentados em gráficos e tabela, os quais foram analisados segundo os conceitos ciberespaço, interconexão, comunidades virtuais e inteligência social, todos de Pierre Lévy. Salienta-se que a interconexão é compreendida a partir de um contínuo sem fronteira, possibilitando a diversidade de uma comunicação interativa. Já as comunidades virtuais, por sua vez, “são construídas sobre as afinidades de interesses, de conhecimentos, sobre projetos mútuos, em um processo de cooperação ou de troca, tudo isso independentemente das proximidades geográficas e das filiações institucionais”. Por fim, a inteligência coletiva aperfeiçoa os saberes e acelera o aprendizado de seus membros, potencializando o desenvolvimento da inteligência coletiva, que “seria sua perspectiva espiritual, sua finalidade última”^(13)^.

## RESULTADOS

As publicações nas redes sociais do Centro de Memória da Escola de Enfermagem da UFMG resultaram em uma média de 6,79 postagens de feed e 1,42 Reels no Instagram, dados referentes aos anos de 2020 e 2021. Para esse mesmo biênio, obteve-se média de 25,54 comentários nas postagens de feed e 512,17 visualizações nos Reels. Quanto ao *Youtube*, observa-se uma média de 1,58 postagens, com 2164,08 visualizações e 101,58 curtidas (Tabela 1).

**Tabela 1:** Caracterização das Publicações realizadas pelo Centro de Memória da Escola de Enfermagem da Universidade Federal de Minas Gerais, Belo Horizonte, Minas Gerais, Brasil, 2021

	Média (IC-95%)	Desvio padrão	Valor de *p* ^*^
Instagram - Postagens de feed			
Nº de postagens	6,79(4,55-9,04)	5,32	0,398
Nº de curtidas	268,50(177,52-359,48)	215,45	0,268
Nº de comentários	25,54(16,57-34,52)	21,25	0,209
Instagram - Reels			
Nº de postagens	1,42(0,51-2,32)	2,15	0,001
Nº de visualizações	512,75(93,95-931,55)	991,80	0,001
Nº de comentários	11,04(1,71-20,37)	22,10	<0,001
Youtube			
Nº de Postagens	1,58(0,52-2,65)	1,68	0,062
Nº de Visualizações	2164,08(-592,88-4921,05)	4339,15	<0,001
Nº de Comentários	3,33(-1,00-7,66)	6,81	<0,001
Likes	101,58(-12,71-215,87)	179,88	<0,001

* *Teste Shapiro-Wilk, ao nível de 5%.*

Observa-se que o número de postagens de feed produzidas e publicadas no Instagram ao longo dos anos de 2020 e 2021 não foi uniforme e variou ao longo dos meses. Pois, apresentou-se maior contagem no período de maio a julho de 2021. Nesse mesmo período, tem-se o crescimento do número de curtidas e comentários (Figura 1).

**Figura 1:** Trajetória entre as contagens das postagens de feed do Instagram que foram realizadas pelo Centro de Memória da Escola de Enfermagem da Universidade Federal de Minas Gerais, Belo Horizonte, Minas Gerais, Brasil, 2021

Na análise bivariada, o coeficiente de correlação de Pearson apontou que as curtidas e os comentários apresentaram correlação positiva com as postagens de feed, r = 0,958 e r = 0,905, respectivamente.

Quanto às postagens de Reels do Instagram, observa-se que entre os meses de agosto de 2020 e maio de 2021 não houve nenhuma produção. O mês de novembro de 2021 foi o de maior quantidade de postagem (7), contudo agosto de 2021 foi o de maior número de visualizações (3611) e comentários (78) (Figura 2). O coeficiente de correlação de Pearson foi positivo entre as postagens de Reels e as visualizações (r = 0,979), bem como os comentários (r=0,940).

**Figura 2:** Análise da trajetória entre as contagens das postagens do Instagram-Reels realizadas pelo Centro de Memória da Escola de Enfermagem da Universidade Federal de Minas Gerais, Belo Horizonte, Minas Gerais, Brasil, 2021

Nesse ínterim, os vídeos produzidos pelo CEMENF para o *Youtube* foram elaborados em maior número em junho de 2021(5), contudo em julho de 2021, apresentaram maior número de visualizações (12361), comentários (21) e *likes* (481) (Figura 3). Os vídeos do *Youtub*e tiveram correlação positiva com as visualizações (r=0,984), comentários (r=0,802) e curtidas (r=0,984).

**Figura 3:** Análise da trajetória entre as contagens das postagens do Youtube realizadas pelo Centro de Memória da Escola de Enfermagem da Universidade Federal de Minas Gerais, Belo Horizonte, Minas Gerais, Brasil, 2021

Logo, as evidências possibilitaram a construção de o desenho imagético que representa a conformação das redes sociais do CEMENF no ciberespaço, à luz dos conceitos de Pierre Levy (Figura 4).

**Figura 4:** Representação da conformação das redes sociais do Centro de Memória da Escola de Enfermagem, da Universidade Federal de Minas Gerais no ciberespaço, Belo Horizonte, Minas Gerais, Brasil, 2021

## DISCUSSÃO

A confecção e a divulgação de conteúdos históricos da enfermagem nas redes sociais contribuem para a conformação do CEMENF no ciberespaço, entendido na perspectiva da prática de comunicação interativa, recíproca, comunitária e intercomunitária^(13)^. Essa conformação se deu pela diversidade de publicações produzidas pelo CEMENF em suas diferentes redes sociais, por meio das postagens de feed e Reels no Instagram e dos vídeos no YouTube.

Percebe-se que o alcance e o engajamento obtido com as publicações, mediante visualizações, curtidas e comentários, constituem o ciberespaço como compreendido por Pierre Levy^(13)^, que o descreve como um mundo virtual vivo, heterogêneo e no qual é possível a participação e a contribuição de cada pessoa. Inserir-se nesse ciberespaço reafirma o compromisso e o desafio do CEMENF não somente com o campo da comunicação museológica, como também com o compartilhamento da história da enfermagem, seja do âmbito local, nacional ou internacional, e para além dos muros institucionais.

Vale enfatizar que o emprego do ciberespaço no campo educacional pode ocorrer pelo uso das tecnologias educacionais digitais. Essas tecnologias, quando utilizadas no ensino de história da enfermagem, incentivam a aprendizagem e reconfiguram esse ensino^(14)^. Ressalta-se que as redes sociais do CEMENF se tornam uma opção de um campo interativo de aprendizagem histórica e de comunicação assíncrona. Ademais, rompem com as formas mais tradicionais de apresentar e de socializar os conteúdos históricos, que podem ser invisíveis para a geração atual de estudantes^(14,15)^.

No contexto de rompimento com os métodos tradicionais, os museus e os centros de memórias apresentam-se como espaços para um processo ativo de ensino-aprendizagem pela articulação dos fatos históricos e atuais, possibilitando uma reflexão crítica, bem como o fortalecimento e o desenvolvimento da identidade profissional^(16)^. Assim, as redes sociais do CEMENF são meios para dar visibilidade a esse espaço, formado por prédio histórico, acervo de indumentárias, acervo documental, iconográfico, dentre outros. Ademais, acredita-se que contribui para o despertar em conhecê-lo, seja mediante visita física ou virtual, denominada visita guiada.

Com efeito, observa-se que as publicações do Instagram, em feed e Reels, e do YouTube, embora apresentem variações ao longo dos meses no período estudado, revelam correlação positiva com curtidas, comentários e visualizações. Essa variação é decorrente do fato de toda a produção dos conteúdos serem feitas por alunos de graduação e pós-graduação em enfermagem, que aliam a construção desses conteúdos de comunicação museológica e de história da enfermagem às atividades curriculares acadêmicas.

Apesar disso, os dados apontam que as redes sociais do CEMENF mantêm a interconexão, estabelecendo o contato e a comunicação interativa com os usuários dessas redes e, assim, construindo-se como espaço envolvente de interação sobre a história da enfermagem mineira, brasileira e internacional. Logo, a interconexão é um dos princípios que orientou o crescimento do ciberespaço e é ela que concebe um contato global^(13)^.

Ademais, na medida em que os dados apresentados apontam o alcance e o engajamento das publicações pelas redes sociais do CEMENF, delineia-se a criação de comunidades virtuais, que compartilham conhecimentos e impressões acerca de interesses comuns, nesse caso, conhecimentos históricos da enfermagem. Essa possibilidade de agregar e de favorecer as interações advindas com as publicações aproximam as pessoas que antes, de acordo com Levy, estavam isoladas ou sem contatos regulares e que agora usufruem de um espaço contínuo de encontro e trocas^(13)^. Além disso, estimulam o aprimoramento da comunicação museológica do CEMENF com o público.

O filósofo Pierre Lévy conceitua a inteligência coletiva como “[...] uma inteligência distribuída por toda parte, incessantemente valorizada, coordenada em tempo real, que resulta em uma mobilização efetiva das competências”^(1)^. O desenvolvimento de tais competências e habilidades nos indivíduos a partir da utilização das tecnologias da informação e comunicação, e quando utilizadas em prol da coletividade, refletem sua essência e relevância^(1)^. Nesse sentido, a utilização do ciberespaço pelo CEMENF pode ser considerada uma estratégia inovadora para construção e produção de conhecimento próprio da enfermagem (e da saúde), uma vez que representa uma reestruturação do ensino e da forma de aprendizagem para a classe da enfermagem.

Por conseguinte, o CEMENF, ao explorar o ciberespaço como ferramenta de alcance de público, socializa a história da enfermagem, além de demonstrar a relevância e a cientificidade da enfermagem. Assim, almeja-se contribuir para a formação de sujeitos críticos, conscientes da história da sua profissão, dotados de uma identidade sólida e capazes de atuarem em prol da valorização social da enfermagem.

Para tanto, o CEMENF apresenta como desafio a constância na produção e publicação de conteúdos para suas redes sociais, de modo a constituir-se como espaço contínuo e permanente de comunicação e conhecimento sobre a história da enfermagem, favorecendo a interconexão, sua consolidação como comunidade virtual e a construção da inteligência coletiva nesse espaço amplo, o ciberespaço. Desse modo, reitera-se a imprescindibilidade das interconexões que fundamentam o ciberespaço em prol de viabilizar a comunicação museológica na atual era da comunicação midiática.

### Limitações do Estudo

A conta do Instagram do CEMENF é uma conta comercial e seus vídeos do YouTube estão hospedados no canal da Escola de Enfermagem da UFMG. Apesar disso, o estudo teve como limitação o não acompanhamento das métricas com mais precisão pelo Painel Profissional, antigo Instagram Insigths e YouTube Analitics. Esse acompanhamento permitiria verificar o perfil do público e os principais insights sobre as contas alcançadas.

### Contribuições para a Área

O Ciberespaço, quando utilizado de forma crítica e reflexiva, pode ser um potente instrumento de aproximação de museus universitários com a sociedade e com a difusão de projetos de extensão e de pesquisa. Desse modo, esse estudo contribui como um incentivo para que outros Centros de Memória possam traçar objetivamente alternativas e caminhos em prol de aumentar as possibilidades de acesso à cultura e à informação, valorizando a enfermagem como ciência e profissão.

## CONCLUSÕES

Em síntese, o estudo mostra como o ciberespaço pode se configurar como importante ferramenta para potencializar o alcance e o engajamento de públicos diversos à cultura e ao conhecimento em saúde, além de abrir oportunidades de diálogo profícuo entre os diversos saberes, considerando-os e somando-os para uma construção dialógica da inteligência coletiva, conforme proposto por Pierre Lévy.
